# Job resources, job demands, and their relationships with thriving, burnout, and work-life conflict among Swedish nursing staff

**DOI:** 10.1186/s12912-026-04693-x

**Published:** 2026-04-30

**Authors:** Karin Myrberg, Annica Björkman, Maria Engström

**Affiliations:** 1 Centre for Research and Development, Uppsala University/Region Gävleborg, Gävle, Sweden; 2 Faculty of Health and Occupational Studies, Department of Caring Sciences, University of Gävle, Gävle, Sweden

**Keywords:** Psychosocial work environment, Nursing, Work-life conflict, Job resources, Working conditions, Staff well-being

## Abstract

**Background:**

Nursing staff working in healthcare are exposed to high job demands, leading to turnover, stress, and decreased motivation. Work-life balance, empowered structures, and thriving have been shown to contribute positively to their psychosocial work environment. This study examined the relationships between job demands, job resources, and the outcomes of thriving, burnout, and work-life conflict among nursing staff.

**Methods:**

A cross-sectional descriptive correlational design was employed, involving 192 nursing staff within somatic in-patient care. Data were collected using the Copenhagen Psychosocial Questionnaire, the Thriving scale, and the Conditions of Work Effectiveness Questionnaire II. Descriptive statistics, Pearson’s correlation coefficients, and multiple regression analyses were conducted to assess the relationships between job demands, job resources, and outcomes.

**Results:**

Job resources, quantified as structural empowerment, showed a positive relationship with thriving and negative relationships with both burnout and work-life conflict. Among job demands, quantitative demands, emotional demands, and work pace demonstrated negative relationships with thriving and positive relationships with burnout and work-life conflict. All resources and demands remained statistically significant in multiple regression models except for emotional demands and work pace on thriving. Regarding interaction effects, structural empowerment moderated the relationship between work pace and both thriving and burnout but did not moderate the relationship with work-life conflict. The interaction between structural empowerment and emotional demands was also statistically significantly positively related to thriving.

**Conclusion:**

The findings of this study underscore the value of adopting strategies that enhance access to empowering job resources, through both top-down and bottom-up approaches, to improve staff well-being. Recognizing and addressing the complex relationships between job demands, resources, and staff outcomes is important for meeting the diverse needs and staffing challenges in healthcare settings.

## Introduction

The psychosocial work environment is widely recognized as one of the most critical aspects of workplace conditions. Research has found it to be closely related to staff wellbeing and the quality of care [1-3]. A significant proportion of nursing staff in the healthcare sector are exposed to several demands at work, leading to consequences such as high turnover rates [4], increased burnout [2] and stress [5]. Balancing professional responsibilities with personal life demands, including leisure, family, and other non-work activities, is referred to as work-life balance [6]. Most hospital nurses report difficulties achieving this balance, often experiencing interference between their work and personal lives [7]. This interference is conceptualized as work-life conflict, defined as a form of interrole conflict in which the general demands, time devoted to, and strain created by the job negatively affect family responsibilities [8, p. 5]. Factors such as role ambiguity, unclear job responsibilities, and role conflict between professional and personal demands are associated with increased stress and are known to exacerbate work-life conflict [9].

Like many other countries, the Swedish healthcare system faces challenges with severe and uneven staff shortages, sometimes referred to as the nursing workforce crisis [10]. Research suggests that a significant number of nurses are contemplating leaving their positions or the profession altogether due to inadequate working conditions and employment terms [11, 12]. High work-life conflicts, low thriving, and burnout have all been found to be related to nurse turnover intentions [5]. Resources in the work environment that ease demands in healthcare—thereby improving thriving, and reducing burnout and work-life conflicts—are therefore of great importance.

### Theoretical framework

Two theories and one model guided the present study. The Job-Demands Resources (JD-R) theory, developed by Demerouti et al. [13], is a well-known, comprehensive, and frequently used theory in a work context. The theory posits that work conditions can be classified into two categories: job demands and job resources. Job demands are aspects that require sustained physical or psychological effort (both hindering and challenging). In contrast, job resources refer to aspects that help achieve work goals, foster personal growth, and enhance motivation. According to the JD-R theory, work resources mitigate the impact of demands and increase staff well-being and effectiveness. Conversely, a work environment with high demands and low resources increases the risk of burnout [2]. So far, outcomes such as thriving and work-life conflict have not been extensively investigated in relation to the JD-R theory. One earlier study found a buffering effect of resources on demands, thereby improving work-life balance [14]. Another study [2] found relationships between demands and thriving as well as resources and thriving, but no buffering effect.

In Kanter’s theory of structural empowerment [15], an empowering structure is described as high/good access to information, resources, and support to accomplish tasks within an organization. Structural empowerment refers to the degree to which employees perceive they have access to and can utilize these structures within their workplace [16]. These structures can be seen as the resources mentioned in the JD-R theory and they directly enhance organizational outcomes [17]. In addition, she highlights formal (a visible and central job within the organization) and informal power (networks within and outside the organization that facilitate work) as central in the theory. In healthcare settings, structural empowerment has been shown to influence nurses’ intentions to leave the profession [18], turnover [12], thriving [1, 19], work-life balance [20], stress [19, 21], and burnout [22]. Moreover, an empowered environment is essential for achieving efficient work, delivering higher-quality care [23], and increasing patient satisfaction [24].

Spreitzer’s Socially Embedded Model of Thriving at Work [25] posits that learning and vitality are essential for employees to thrive, leading to positive health and development. Thriving at work is a positive psychological state where employees feel energized and are learning. This occurs due to supportive social factors (open information sharing, and a trusting, respectful environment) and proactive behaviours (such as focusing on tasks, exploring new ideas, and mindful interactions). These elements together enhance employees’ well-being [25]. According to the JD-R theory and structural empowerment theory, job demands and resources, including structural empowerment have been linked to thriving [18]. Additionally, JD-R and structural empowerment have been linked to burnout [22].

In summary, nurse thriving is central in healthcare, an information-intensive workplace where staff need to continuously learn to stay up to date with the latest research and guidelines. Despite its importance, research on thriving within the nursing field remains limited, although there has been an upward trend in recent years. Additionally, work-life balance is crucial, as it has been shown to be related to turnover intentions in professions such as registered nurses [26] where there is already a significant shortage. The relationships between work-life conflict, structural empowerment, and the JD-R theory have been studied to a lesser extent. High job demands and insufficient access to resources at work may negatively affect staff members’ personal lives, leading to conflicts and stress in the work-nonwork interface [27]. These adverse effects highlight the need to empower staff to improve their individual resources and performance [14]. In Martinez-Corts et al.’s [27] conclusions, they argue for empowering staff and thereby strengthen their individual resources and performance. Given the current nursing workforce situation [10], there is a need for a more comprehensive examination of these factors. This study aims to contribute to this emerging discussion, with a specific focus on thriving and work-life conflict.

### Aim and research questions

Thus, based on earlier research, the JD-R theory, Kanter’s theory, and, for one of the outcomes, Spreitzer’s model of thriving at work, the aim is to study the relationships between job demands (quantitative demands, emotional demands, work pace), job resources (structural empowerment), and three outcomes: thriving, burnout, and work-life conflict among nursing staff working in somatic in-patient care.

#### Hypotheses (H)

##### H1

There is a positive relationship between job resources (measured as structural empowerment) and thriving.

##### H2

There is a negative relationship between job resources (measured as structural empowerment) and burnout.

##### H3

There is a negative relationship between job resources (measured as structural empowerment) and work-life conflicts.

##### H4

There is a negative relationship between job demands (measured as quantitative demands, emotional demands and work pace) and thriving.

##### H5

There is a positive relationship between job demands (measured as quantitative demands, emotional demands and work pace) and burnout.

##### H6

There is a positive relationship between job demands (measured as quantitative demands, emotional demands and work pace) and work-life conflict.

##### H7

Job resources have a buffering effect on job demands, which is related to an increase in a) thriving, and a decrease in b) work-life conflicts and c) burnout.

## Methods

### Study design, sample and setting

A cross-sectional descriptive correlational design was used. The study was part of a larger project, ‘Nursing Acuity Measurements in Somatic In-patient Care’ (NCT06035705; Grant: Afa Insurance 230,157). Data for this study were collected at baseline, independently of the intervention, and included registered and assistant nurses working in somatic inpatient care (*n* = 192) in late 2023. Guidelines from Strengthening the Reporting of Observational Studies in Epidemiology (STROBE) Statement for observational studies were followed [28].

The decentralised Swedish healthcare system is divided into 21 self-governing regions. This study took place in a Mid-Sweden region, with about 285,000 residents and 6,000 employees in the healthcare sector. To obtain a nursing license in Sweden, you must complete a 3-year bachelor’s level nursing program. Since 2023, the title of assistant nurse is protected, requiring a formal education of three years in upper secondary school (or equivalent). Certificates are issued by the Swedish Board of Health and Welfare.

About 400 registered nurses and assistant nurses working in 12 somatic in-patient wards across three hospitals in the region were asked to participate between November and December 2023. The only inclusion criterion was that the individuals must have been employed in the unit for more than one month. Employees who were absent for various reasons during the questionnaire period were automatically excluded, as it was sent to their work email addresses.

### Data collection

All nursing staff members at the 12 wards received an email with a link to a web-based questionnaire, along with an introduction and information letter about the research project. Each questionnaire was open for 3 weeks and included two reminders. Consent was given if the staff members agreed to complete the questionnaire. In the study information, participants were assured that their responses would remain confidential.

### Instruments

The questionnaire was based on a selection of common and validated questionnaires: parts of the Swedish versions of the Copenhagen Psychosocial Questionnaire (COPSOQ III) [29], Thriving scale [30], and the Conditions of Work Effectiveness Questionnaire II (CWEQ-II) [31]. For an overview of the scales and items used, please see Table 1.

**Table 1:** Overview of the scales and items presented in this paper

Assessment	Job demands	Burnout	Work-life conflicts	Structural empowerment	Thriving
Instruments	Copenhagen Psychosocial Questionnaire (COPSOQ)			Conditions of Work Effectiveness Questionnaire (CWEQ-II)	Thriving scale
References	Pejtersen et al., 2020; Swedish version by Berhelsen et al. 2020			Laschinger et al. 2012; Swedish version by Engström et al. 2011	Porath et al. 2012 Swedish version by Engström et. al. 2025.
Description	*A comprehensive tool designed to assess psychosocial factors at work, including stress, job satisfaction, and work-life balance*			*Measures structural empowerment by assessing employees’ access to opportunity, information, support, and resources in their work environment.*	*Assesses the psychological state of thriving, characterized by a sense of vitality and learning.*
Scales, indices and items	*COPSOQ III * *(5-point scales, with the answers being ‘always’, ‘often’, ‘sometimes’, ‘rarely’, ‘never/almost never’)* _Quantitative demands (3 items) _Work pace (2 items) _Emotional demands (*2 items, since the item “Is your work emotionally demanding?” was missing by mistake in our data collection)*	*COPSOQ III * *(5-point scales, with the answers being ‘always’, ‘often’, ‘sometimes’, ‘rarely’, ‘never/almost never’)* _Burnout (3 items)	*COPSOQ III * *(5-point scales, with the answers being ‘to a very high degree’, ‘to a high degree’, ‘to some extent’, ‘to a low degree’, ‘to a very low degree’)* Work life conflict (3 items) *For COPSOQ dimension scores are the mean of item scores (with reverse scoring when needed)*	*CWEQ-II* *(5-point scales, with the answers ranging from 1=’none’ to 5=’a lot’)* _Opportunities (3 items) _Information (3 items) _Support (5 items) _Resources (3 items) _Job activities (formal power) (3 items) _Organization relationships (informal power) (4 items) *An overall empowerment score is calculated by summing the six subscales.*	*Thriving scale* *(7-point scale, with the answers ranging from 1=’strongly disagree’ to 7=’strongly agree’)* _Vitality (5 items) _Learning (5 items) *The total score for thriving was used in the present study.*

To assess staff perceptions of psychosocial working conditions and demands (such as burnout, work-life conflicts, emotional demands, work pace demands and quantitative demands), we utilized five subscales from the Swedish version of the COPSOQ, which has demonstrated good psychometric properties [29]. Each scale included between two to four items. The COPSOQ incorporates concepts from several traditional theories of psychosocial working conditions, such as the JD-R model.

The CWEQ-II, developed by Laschinger [32] and based on Kanter’s theory of structural empowerment [15] was used to measure the staffs’ access to empowering structures such as opportunities for growth, support, and access to information and resources within the organization. The questionnaire includes subscales that measure formal power, informal power, and perceived access to work empowerment structures. The instrument has demonstrated good psychometric properties [31].

To assess staff’s perception of thriving, the Thriving at work scale [30] was administered. The scale consists of the two factors ‘vitality’ and ‘learning’. Previous research on the Swedish version has shown internal consistency with α > 0.80 for both the factors and the overall score [24] and construct validity is also supported [1].

### Data analysis

Data were analysed in IBM SPPSS Statistics version 29 with statistical significance defined as *p* < 0.05 for all analyses. The outcome variables were thriving, burnout and work-life conflicts while predictors were job demands (quantitative demands, emotional demands, work pace), and job resources (structural empowerment), age and sex controlled for.

The initial step involved calculating descriptive statistics to summarise respondent characteristics. This included presenting categorical variables as frequencies and percentages, as well as mean and standard deviation for continuous variables.

Pearson’s correlation coefficients were computed to assess the strength and direction of the relationships between job demands, job resources, and the outcomes. Following the bivariate analysis, three separate multiple linear regression analyses were conducted to determine the relationships between job demands, job resources and the outcomes of thriving, burnout, and work-life conflict. This approach was chosen as we were interested in which variables would be significant using a model with several predictors, including interaction terms (demands × resources). For the regression analyses, we followed the guideline of 50 + 8 × number of predictors [8], meaning at least 106 participants were needed for our models. As this study uses baseline data from a larger intervention project, the final sample size depended on the available data. Age and sex were examined as covariates. Bivariate correlation analyses of age and the outcome were conducted and sex differences in outcomes were conducted using Student’s t test for independent samples. Covariates that were significantly associated with the outcomes in the bivariate analyses were subsequently entered into the regression models. Thereafter interactions were added (demands × resources) one by one to the models. Standardized coefficients (Beta) and *p*-values were reported to evaluate the significance and impact of each predictor variable.

R-squared (R^2^) and adjusted R-squared (Adj R^2^) values indicate the proportion of variance in the dependent variables explained by the independent variables. Multicollinearity was tested with variance inflation factor (VIF), ensuring that no significant multicollinearity issues were present among the predictors, as expected they were high when interaction terms were added while for the other models all VIF values were below 2.5. Visual inspections of residuals showed no serial deviations from the normal distribution.

### Research ethics

The study was approved by the Swedish Ethical Review Authority Dnr 2023–03688-01. All participants received written study information, completing and returning the questionnaire indicated tacit informed consent.

## Results

### Respondent characteristics

A total of 192 respondents completed the questionnaire, yielding a response rate of 48%. There was a slight majority of assistant nurses (56.2%) compared to registered nurses (43.8%), of whom 3.6% were specialized. Most participants were female (88.5%), while 11% were male and 0.5% preferred not to disclose their sex. The mean age was 40.1 years (SD 12.5, median 38), and participants had, on average, 10.1 years of professional experience (SD 11.3, median 6). The majority (83.9%) worked full-time, while 16.1% worked part-time.

### Descriptive statistics and relationships between study variables

Mean values and bivariate relationships between the study variables among nursing staff working in somatic in-patient care are presented in Table 2. All hypothesized relationships were statistically significant, with the strongest negative correlation between work pace and work-life conflict (*r* = −.55), and the strongest positive correlation between structural empowerment and thriving (*r* = .64). In the analyses of covariates, women had higher mean burnout scores than men, with a mean score of 56.3 (SD = 22.1) for women and 42.5 (SD = 25.8) for men (*p* = .009) and thereby included in the multiple linear regression models. For thriving and work-life conflict, sex differences were non-significant and thus not included in the multiple linear regression models. Age demonstrated statistically significant relationships on all outcomes in bivariate analyses. However, the level of education (registered nurses vs. assistant nurses) did not show a statistically significant relationship with any of the outcomes. Findings will be further explored in the context of the hypotheses in the subsequent section.

**Table 2:** Relationships between study variables and descriptive statistics

	Mean (SD)	Thriving	Burnout	Work-life Conflict
*Mean (SD)*		*5.2 (0.9)*	*54.9 (22.9)*	*44.7 (28.8)*
*Structural Empowerment*	*19.6 (3.5)*	0.644^***^	−0.422^***^	−0.505^***^
*Quantitative Demands*	*48.6 (16.8)*	−0.379^***^	0.502^***^	0.546^***^
*Emotional demands*	*53.8 (21.1)*	−0.391^***^	0.513^***^	0.522^***^
*Work pace*	*64.3 (20.5)*	−0.381^***^	0.550^***^	0.590^***^
*Age*	*40.1 (12.5)*	0.150*	−0.264***	−0.238***

Abbreviation SD standard deviation, *p < 0.05, **p < 0.01, ***p < 0.001

Tables 3, 4, and 5 present the results of the multiple linear regression analyses for the three outcomes: thriving, burnout, and work-life conflict. Each table displays standardized coefficients (Beta), *p*-values, R^2^, and adjusted R^2^, including the effects of demographic variables (age, sex) where applicable. In each table, model 1 includes only the predictors without interaction terms (i.e. only the main effects). In models 2 and 3, interaction terms between job demands and structural empowerment were added one at a time; only interactions with *p*-values less than 0.1 are reported in the tables.

**Table 3:** Multiple linear regression models of thriving with job demands and structural empowerment

Thriving	Model 1		Model 2		Model 3	
	β	p-value	β	p-value	β	p-value
R^2, /^Adj R^2^	0.462/0.448		0.493/0.476		0.488/0.471	
*p*-value model		<0.001		<0.001		<0.001
Structural empowerment	**0.549**	<0.001	0.088	0.558	0.225	0.066
Quantitative demands	**−0.157**	0.021	−0.116	0.086	−0.122	0.071
Emotional demands	−0.074	0.281	−0.079	0.236	**−0.908**	0.001
Work pace	−0.032	0.674	**−0.968**	<0.001	−0.048	0.520
Age	0.053	0.335	0.067	0.211	0.044	0.417
Sex	-		-		-	
SE*Work pace			**0.874**	<0.001		
SE*Emotional demands					**0.772**	0.003
VIF highest	2.0 Work pace		30.5 Work pace		28.5 Emotional demands	

*Note. Model 1 includes only the predictors without interaction terms (main effects only). Model 2 and Model 3 each include one interaction term between structural empowerment and job demands (work pace or emotional demands), entered one at a time. Bold values indicate statistically significant results. SE = Structural empowerment, β = standardized coefficient. VIF = variance inflation factor. The rise in VIF is expected when terms already measured are included again as part of interaction variables. “–” indicates non-significant results in the univariate analyses and thus not included in the multiple linear regression analyses*

**Table 4:** Multiple linear regression models of burnout with job demands and structural empowerment

Burnout	Model 1		Model 2	
	β	*p*-value	β	*p*-value
R^2, /^Adj R^2^	0.454/0.436		0.469/0.449	
*p*-value model		<0.001		<0.001
Structural empowerment	**−0.176**	0.005	0.149	0.334
Quantitative demands	**0.189**	0.007	**0.157**	0.026
Emotional demands	**0.211**	0.003	**0.897**	0.003
Work pace	**0.232**	0.003	**0.214**	0.002
Age	**−0.160**	0.004	**−0.169**	0.002
Sex	−0.064	0.256	−0.076	0.173
SE*Work pace			**−0.619**	0.023
VIF highest	2.0 Work pace		30.9 Work pace	

*Note. Model 1 includes only the predictors without interaction terms (main effects only). Model 2 includes one interaction term between structural empowerment and work pace, entered in addition to the main effects. Bold values indicate statistically significant results. SE = Structural empowerment, β = standardized coefficient. VIF = variance inflation factor. The rise in VIF is expected when terms already measured are included again as part of interaction variables. “–” indicates non-significant results in the univariate analyses and thus not included in the multiple linear regression analyses*

**Table 5:** Multiple linear regression models of work-life conflict with job demands and structural empowerment

Work-Life Conflict	Model 1		Model 2	
	β	*p*-value	β	*p*-value
R^2, /^Adj R^2^	0.519/0.506		0.528/0.513	
*p*-value model		<0.001		<0.001
Structural empowerment	**−0.259**	<0.001	−0.007	0.961
Quantitative demands	**0.236**	<0.001	**0.213**	0.001
Emotional demands	**0.163**	0.013	**0.166**	0.011
Work pace	**0.244**	<0.001	**0.756**	0.007
Age	**−0.128**	0.014	**−0.136**	0.009
Sex	-		-	
SE*Work pace			−0.479	0.059
VIF highest	2.0 Work pace		27.6 Work pace	

*Note. Model 1 includes only the predictors without interaction terms (main effects only). Model 2 includes one interaction term between structural empowerment and work pace, entered in addition to the main effects. Bold values indicate statistically significant results. SE = Structural empowerment, β = standardized coefficient. VIF = variance inflation factor. The rise in VIF is expected when terms already measured are included again as part of interaction variables. “–” indicates non-significant results in the univariate analyses and thus not included in the multiple linear regression analyses.*

### Relationship between job resources and job demands with thriving (H1 and H4)

The bivariate correlation analysis revealed, statistically significant positive relationship between job resources (measured as structural empowerment) and thriving, supporting H1. Additionally, statistically significant negative relationships were found between job demands (quantitative demands, emotional demands, and work pace) and thriving, supporting H4 (see Table 2).

The multiple linear regression analysis (Model 1, Table 3) confirmed that structural empowerment had a statistically significant positive relationship with thriving, and quantitative demands a statistically significant negative relationship with thriving. For emotional demands and work pace the results were non-significant. The model explained 46.1% of the variance in thriving (see Table 3).

### Relationship between job resources and job demands with burnout (H2 and H5)

The bivariate correlation analysis revealed a statistically significant negative relationship between job resources (measured as structural empowerment) and burnout, thus supporting H2. The bivariate analysis also showed statistically significant positive relationships between job demands (quantitative demands, emotional demands, and work pace) and burnout, supporting H5 (see Table 2).

The multiple linear regression analysis (Model 1, Table 4) confirmed that structural empowerment had a statistically significant negative relationship with burnout. In addition, all three job demands, quantitative demands, emotional demands, and work pace, had statistically significant positive relationships with burnout. The model accounted for 45.4% of the variance in burnout (see Table 4).

### Relationship between job resources and job demands with work-life conflicts (H3 and H6)

The bivariate correlation analysis revealed a statistically significant negative relationship between job resources (measured as structural empowerment) and work-life conflict, thus supporting H3. The bivariate correlation analysis showed statistically significant positive relationships between job demands (quantitative demands, emotional demands, and work-pace) and work-life conflict, supporting H6 (see Table 2).

The multiple linear regression analysis (Model 1, Table 5) confirmed that structural empowerment had a statistically significant negative relationship with work-life conflict. All three job demands were positively related to work-life conflict. The model accounted for 53% of the variance in work-life conflict (see Table 5).

### Buffering effect of job resources on job demands: impact on thriving, burnout, and work-life conflicts (H7)

To test the buffering effect of job resources on job demands, interaction terms (job resources × job demands) were added one by one to the regression models. The results revealed statistically significant interactions between work pace and structural empowerment in relation to thriving (Table 3, Model 2) and burnout (Table 4, Model 2). The interaction between emotional demands and structural empowerment was also significant for thriving (Table 3, Model 3). The interaction between work pace and structural empowerment was not significant for work-life conflict (Table 5, Model 2, *p* = .098). For other interactions not mentioned here, such as quantitative demands × resources, the results were non-significant.

## Discussion

Our results demonstrate that job resources, here quantified as structural empowerment, are central to promoting thriving and reducing both burnout and work-life conflict among nursing staff. Conversely, job demands, encompassing quantitative and emotional demands as well as work pace, negatively impact these outcomes. These findings support the hypotheses and align with the JD-R model.

### Thriving at work

Our results show that higher levels of structural empowerment are positively related to nursing staffs’ thriving at work, in line with previous research highlighting the importance of access to opportunities, information, support and power for staff well-being [15, 25, 32]. It is well established that structural empowerment is linked to better nurse job performance [33] and is theorized to foster a sense of meaning in work [31]. According to Kanter, empowerment provides individuals with agency, that is, the ability to get things done within the organization [15]. Structural empowerment has also been related to quality of care as perceived by both staff [16] and patients [24]. Nurses in our sample reported moderate empowerment, with a mean CWEQ-II score of 19.6, aligning well with findings from previous studies on nurse empowerment [1, 33, 34].

Quantitative demands were negatively related to thriving, supporting the idea that excessive workload undermines vitality and learning at work [35]. Although emotional demands and work pace were not significant predictors in model 1, the interaction analyses (Model 2, Model 3) revealed that structural empowerment can buffer the negative effects of both work pace and emotional demands, supporting H7 for thriving. This reflects the evolution of the JD-R model, which not only addresses employee strain but also highlights the workplace conditions that promote thriving [35]. This is consistent with earlier research showing that certain workplace conditions, such as information sharing and feedback, are important for individual thriving [25], and that first-line managers, through their leadership styles, can further enhance nursing staff’s ability to thrive at work [36]. Our findings thus extend the JD-R literature by demonstrating that thriving, a relatively underexplored outcome, benefits from both direct and moderating effects of job resources.

### Burnout

Consistent with previous studies [22, 37], our results demonstrate that job demands are related to increased burnout among nursing staff, while structural empowerment is negatively related to burnout.

All three job demands (quantitative, emotional, and work pace) were significantly related to burnout in the regression model, supporting H5. Structural empowerment was found to buffer the impact of work pace on burnout, indicating that access to empowering resources can mitigate the negative effects of high work intensity. This finding aligns with prior research showing that access to job resources, such as supportive and transformational leadership, positive interpersonal relationships, autonomy, and professional resources, can buffer the negative impact of job demands [14, 37, 38]. However, no significant moderating effect was found for emotional demands or quantitative demands, suggesting that some types of job demands may be more responsive to resource buffering than others. This supports the JD-R theory’s proposition that job resources not only reduce strain but can also offset the impact of demanding work conditions [35]. Although women reported significantly higher levels of burnout than men in both the bivariate and adjusted analyses, this finding underscores the importance of considering gender differences when addressing burnout among nursing staff. Also, consistent with previous literature, addressing burnout is not only critical for the well-being of nursing staff and their employers, but may also be an effective strategy for improving the quality of care [39]. Our findings thus underscore the importance of promoting empowering work environments and ensuring sufficient job resources to help staff manage high demands and reduce the risk of burnout.

### Work-life conflict

Consistent with previous research, indicating that hospital nurses experience greater difficulties balancing work and private life compared to other professions [7], our sample demonstrate higher levels of work-life conflict (mean 44.7) than the Swedish COPSOQ-III reference value (39.7) [29]. All three types of job demand, quantitative demands, emotional demands, and work pace, were positively related with work-life conflict, supporting H6 and highlighting the considerable challenges posed by the demands of somatic in-patient care. In contrast, access to job resources in the form of structural empowerment was significantly related to lower levels of work-life conflict, supporting H3. However, our interaction analyses did not reveal a statistically significant buffering effect of structural empowerment on the relationship between job demands and work-life conflict, suggesting that, while empowering resources help reduce overall conflict, they may not fully offset the negative impact of high demands on work-life balance in this context. These findings are in line with the JD-R theory, which posits that job resources can mitigate, but not always eliminate, the impact of job demands on strain and conflict [35]. It is essential to address work-life conflict to improve the well-being and satisfaction of nursing staff and help retain them in the workforce. Our study suggests that both reducing job demands and promoting structural empowerment can help nurses achieve a healthier balance between work and life.

### Methodological considerations

This study is cross-sectional, which means we cannot draw conclusions about causality. The response rate was 48%, potentially limiting the generalizability of the findings, although this rate is consistent with previous studies [40]. Another limitation concerns the use of self-reported questionnaires, which may affect the accuracy of the findings [41]. Additionally, one item in the COPSOQ III, ‘Is your work emotionally demanding?’, was accidentally missing from the questionnaire. This non-response may have affected the reliability of the emotional demands subscale and could introduce systematic error if the missing item captures aspects of emotional demands not fully represented by the remaining items. As a result, the interpretation of findings related to emotional demands should be made with caution.

It should also be noted that about one-third of respondents in the present study had worked for less than five years. More experienced nursing staff may develop improved coping mechanisms and time-management strategies [42], so the inclusion of a relatively high number of less experienced nurses may have influenced the results. Nevertheless, prolonged exposure to occupational stressors may increase vulnerability to chronic fatigue and burnout, even among experienced staff [43].

Finally, as this study was conducted within a single organization, this may limit the generalizability of the results to other healthcare organizations with varying access to resources. Study strengths include the use of validated instruments, and the inclusion of nursing staff from a variety of in-patient specialities and three different hospitals.

### Implications

It is also important to recognize that high job demands, such as those in in-patient nursing, carry physiological and psychological costs [44]. To promote thriving and reduce burnout and work-life conflict, healthcare organizations should use the JD-R theory to improve access to empowering job resources through both top-down and bottom-up approaches [35]. While job design in healthcare has often focused on management- and HR-led (top-down) strategies, this can overlook individual differences in how resources are perceived and used, even among staff in similar roles [45]. Empowerment is most effective when both organizational structures and individual agency are considered [21].

Rather than concentrating on difficulties and strains, organizations should implement supportive structures and policies that empower nursing staff and foster a developmental work environment where nurses feel valued. At the unit and individual levels, first-line managers and staff should proactively enhance their resources by making positive changes to their work, such as appreciating each other’s contributions, seeking feedback, learning new skills, and supporting new staff in understanding the value of empowerment [46]. Such bottom-up approaches can help address unique needs, foster autonomy, and create a more positive work culture.

Given the ongoing shortage of qualified nurses and high turnover rates [47], understanding and addressing these mechanisms is crucial for effectively meeting the diverse needs and challenges within healthcare settings. Future studies should further explore how specific empowering resources can be effectively implemented and sustained in different healthcare contexts. Longitudinal research could also help clarify causal relationships between job resources, demands, and staff well-being.

## Conclusions

In conclusion, the present study demonstrates that empowering job resources positively relate to thriving and negatively to both burnout and work-life conflicts among nursing staff. In contrast, job demands, including quantitative and emotional demands as well as work pace, negatively relate to thriving and positively to burnout and work-life conflicts. This study adds to the established JD-R theory by demonstrating that job resources are not only related to better outcomes for nursing staff but may also moderate the relationships between job demands and outcomes, potentially making it easier to cope with workplace pressures.

## Data Availability

The summary data can be found in the main document. Aggregated data are available from the corresponding author upon reasonable request. Signing a data sharing agreement will be necessary. Individual data are unavailable due to General Data Protection Regulations (GDPR) and in accordance with the ethics application.
